# A lysate proteome engineering strategy for enhancing cell-free metabolite production^☆^

**DOI:** 10.1016/j.mec.2021.e00162

**Published:** 2021-01-22

**Authors:** David C. Garcia, Jaime Lorenzo N. Dinglasan, Him Shrestha, Paul E. Abraham, Robert L. Hettich, Mitchel J. Doktycz

**Affiliations:** a Biosciences Division, Oak Ridge National Laboratory, Oak Ridge, TN, USA; b Bredesen Center for Interdisciplinary Research, University of Tennessee, Knoxville, TN, USA; c Graduate School of Genome Science and Technology, University of Tennessee, Knoxville, TN, USA; d Chemical Sciences Division, Oak Ridge National Laboratory, Oak Ridge, TN, USA

**Keywords:** Genome engineering, Cell-free metabolic engineering, Cell lysates, In vitro biology, Proteomics

## Abstract

Cell-free systems present a significant opportunity to harness the metabolic potential of diverse organisms. Removing the cellular context provides the ability to produce biological products without the need to maintain cell viability and enables metabolic engineers to explore novel chemical transformation systems. Crude extracts maintain much of a cell’s capabilities. However, only limited tools are available for engineering the contents of the extracts used for cell-free systems. Thus, our ability to take full advantage of the potential of crude extracts for cell-free metabolic engineering is constrained. Here, we employ Multiplex Automated Genomic Engineering (MAGE) to tag proteins for selective depletion from crude extracts so as to specifically direct chemical production. Specific edits to central metabolism are possible without significantly impacting cell growth. Selective removal of pyruvate degrading enzymes resulted in engineered crude lysates that are capable of up to 40-fold increases in pyruvate production when compared to the non-engineered extract. The described approach melds the tools of systems and synthetic biology to showcase the effectiveness of cell-free metabolic engineering for applications like bioprototyping and bioproduction.

## Highlights

• A method of engineering cell-free metabolism in lysates is described.
• Method enables design of cell lysates for enhancing specific metabolic processes.
• Pyruvate consuming enzymes tagged with 6xHis tags have minimal impact on growth.
• Post-lysis pull-down of tagged enzymes enables cell-free pyruvate pooling.
• Lysate engineering strategy permits metabolic states not possible in living cells.

## 1 Introduction

Driven by the prospect of biological systems that can be easily manipulated, the application of synthetic biology tools to *in vitro* environments offers a promising approach to harnessing an organism’s rich metabolic potential (Guterl et al., 2012). Cell-free systems use cytoplasmic components, devoid of genetic material and membranes, as a means of producing complex chemical transformations. While living cells require membranes, growth substrates, and biochemical regulation, *in vitro* systems sidestep these barriers to manipulation and present an opportunity to explicitly define a system for creating novel proteins and metabolites (Shin and Noireaux, 2012). In this way, cell-free metabolic engineering (CFME) can use the organism’s existing biochemical functions and further combine these capabilities with heterologous pathways to produce chemical precursors, biofuels, and pharmaceuticals.

Efforts to engineer cell-free systems have taken different approaches. Ideally, a CFME system would contain a minimal set of components necessary to carry out a desired biochemical process. Previous approaches employed a defined set of purified enzymes for producing high-yielding chemical conversions and have successfully demonstrated a variety of capabilities including chemical production and protein synthesis (Opgenorth et al., 2016; Wang et al., 2012). Constructing complex, multistep pathways require significant development and upfront costs as utilizing purified proteins at scale remains costly (Korman et al., 2017). Further, these purified component systems can exhibit slow catalysis rates, possibly due to the lack of accessory proteins and appropriate protein concentrations capable of improving pathway yield (Korman et al., 2017). Nevertheless, long-running CFME systems that can catalyze multi-step reaction pathways for days have been developed (Opgenorth et al., 2016).

The use of crude cell extracts presents an alternative approach to CFME. Simple cell lysis and minimal fractionation can be rapidly carried out and result in complex enzyme mixtures for a fraction of the cost of purified components (Sun et al., 2013; Kay and Jewett, 2015; Garcia et al., 2019). Crude extract systems derived from both commonly used cell-free model organisms, such as *E. coli* BL21 Star (DE3), or nontraditional strains, such as *Vibrio natriegens*, contain a similar biochemistry to the donor cell and can serve as both prototyping tools for *in vivo* metabolic engineering and as bioproduction platforms (Kay and Jewett, 2015; Hurst et al., 2017; Rollinet al., 2015; Wiegand et al., 2019). Cell-free systems work well for both prototyping and production as CFME can be modularly assembled with lysates enriched for specific enzymes or entire metabolic pathways in order to produce a specific molecule (Krüger et al., 2020). Additionally, their compatibility with chemical reactors and ability to consume low-cost feedstocks have popularized them as potential sources for industrial production (Timm et al., 2016; Tinafar et al., 2019; Lu, 2017). These combined capabilities allow CFME processes to make use of tools from traditional bioproduction platforms while taking advantage of the open and modular nature of cell-free systems.

While environmental variables of a cell-free system can be easily manipulated, the proteomic content of the crude extract is more difficult to engineer. Genetic manipulation of a donor strain can substantially impact its growth and function as a bioproduction system (Garcia et al., 2018). It has been noted previously that simple variations in growth conditions can lead to complex changes in the proteome and significant differences in metabolite flux in the resulting crude extracts (Garcia et al., 2018; Schmidt et al., 2016). Further, specific enzymes can be added or expressed in an extract to further define metabolite production (Guterl et al., 2012). However, removing specific proteins is challenging as gene deletions can affect the growth and global expression of the donor cell. In particular, deletions to central metabolism can be lethal, which severely limits the ability to direct flux from simple carbon sources. The inability to remove specific pathways from CFME reactions poses a significant constraint and limits the use of crude extracts for bioproduction (Kay and Jewett, 2015; Garcia et al., 2018). Tools that allow shaping of the cell-free proteome have been proposed but have not been applied towards the manipulation of cell-free metabolism. Instead, these efforts have focused on improving various single aspects of transcription and translation (Yin et al., 2012; Oesterle et al., 2017). Providing approaches with the ability to modulate the presence of multiple enzymes and specific pathways will be critical in enabling the use of crude extract systems for metabolic engineering applications.

**Fig. 1:**
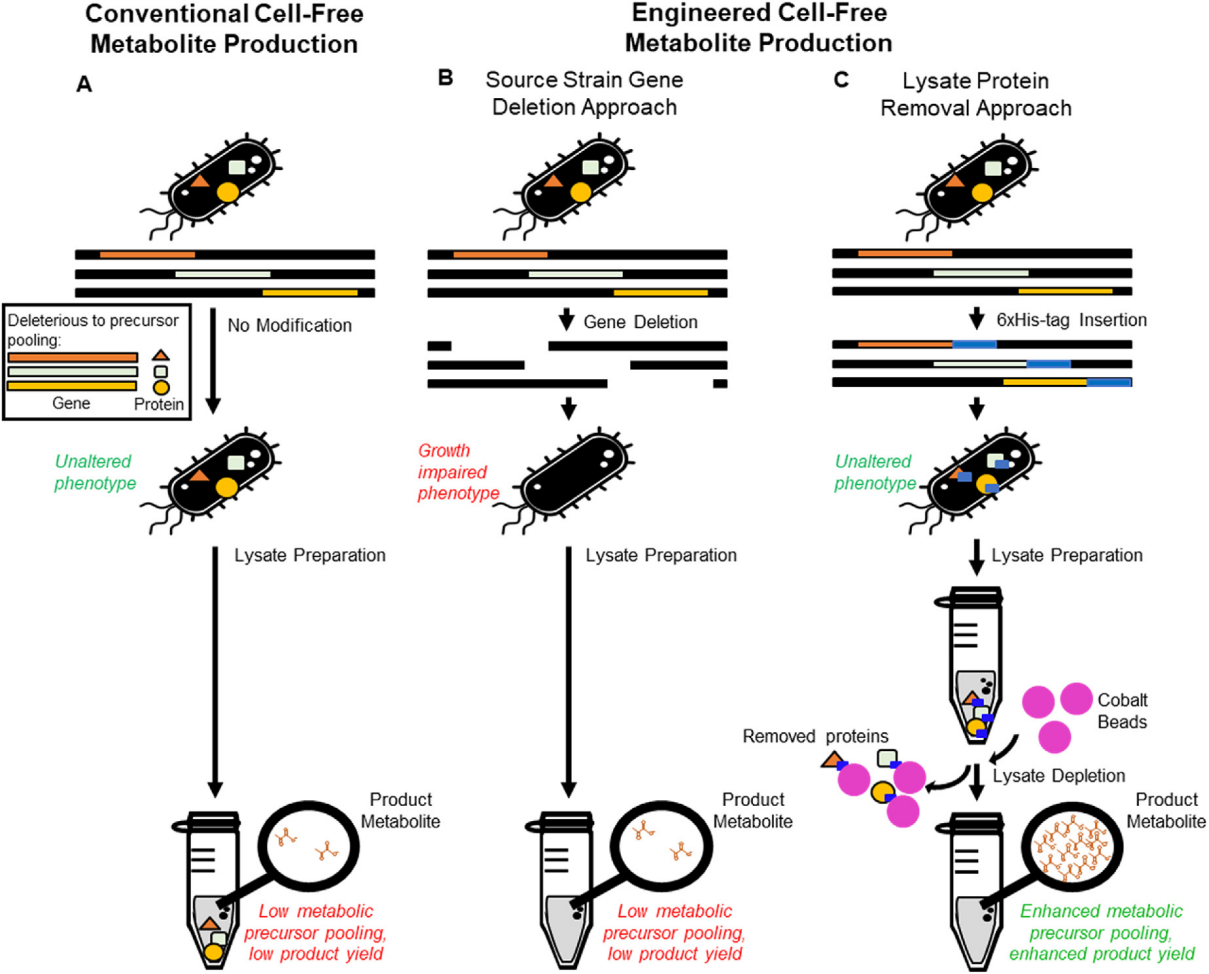
Overview of approaches to preparing lysates for cell-free metabolic engineering. A. Complex metabolism present in E. coli lysates harnessed for cell-free metabolite production can compromise central metabolic precursor yields. B & C. Cell-free metabolic engineering approaches seek to reduce lysate complexity in order to redirect carbon flux and pool central metabolic precursors. B. The standard CFME approach reduces lysate complexity by deleting target genes from the source strain, often resulting in growth impaired or lethal phenotypes due to the inability to remove essential genes. This can require multiple design-build-test cycles. C. The new approach involves engineering source strains to endogenously express recognition sequences, such as 6xHis-tags, into target proteins for subsequent removal from lysates through affinity purification, resulting in minimal to no impact on source strain growth and enhanced pooling of specific metabolic products.

In this work, we describe the use of genome engineering, specifically MAGE, to enable the removal of particular proteins from crude extracts for CFME. 6xHis-tags are incorporated into proteins that are expected to consume pyruvate and are used for the affinity-based depletion of these proteins following cell lysis. Pyruvate, a key connection point in central carbon metabolism, was chosen due to its role linking glycolysis and the Krebs cycle, as well as its relevance as a central precursor for numerous products (Zhang et al., 2016; Maleki and Eiteman, 2017). The use of genome engineering results in a modified proteome capable of producing engineered metabolic phenotypes with minimal impact on the viability of the donor cell (Fig. 1) (Dai and Nielsen, 2015; Rydzak et al., 2017). This general strategy was demonstrated using *E. coli* BL21 Star (DE3) as a chassis due to its prevalent use for cell-free synthetic biology.

## 2 Methods

### 2.1 Generation and validation of genome engineered strains using MAGE

All multiplex allele-specific PCR (MASC-PCR), Sanger Sequencing oligos, and recombineering oligos were created manually and ordered from IDT (Coralville, IA) with standard purification. Each targeting oligo incorporated four phosphorothioated bases on the 5′ terminus. An 18-base CACCATCACCATCACCAT sequence was used to add the 6xHis-tag and directed at either the N- or C-terminus based on previous literature or crystal structure analysis (Lougheed et al., 2014; Halliwell et al., 2001). The pORTMAGE protocol used in this study followed previous work with the exception that growth was carried out in 6 ​mL of Luria-Bertani-Lennox (lbl) cultures in glass tubes with 100 ​mg ​mL^−1^ of carbenicillin, recovery was performed in 3 ​mL of terrific broth with a 1-h incubation time prior to adding 3 ​mL of lbl-carb for outgrowth (Nyerges et al., 2016). Given the significant time required to find accumulated mutations in a single strain, the additive mutations were started from previously found mutations such that Δ1 was used to create Δ2 and so on as per the protocols used in previous studies (Wang et al., 2012). After every 8–12 cycles of MAGE, 30–60 colonies were screened for genome edits using MASC-PCR as detailed previously (Wang et al., 2012). Allelic genotyping was performed using standard primers designed to flank both modified genes. Amplicons were Sanger sequenced to validate the insertion of the 6xHis-tag sequence. Primer sequences used in this study are listed in Supplemental Table 1 and Supplemental Table 2.

### 2.2 Cell-free extract preparation protocol

Following plasmid curing, the cell extracts were prepared from *E. coli* BL21 Star (DE3) grown at 37 ​°C in 2xYPT-G (16 ​g L^-1^ tryptone, 10 ​g L^-1^ yeast extract, 5 ​g L^-1^ NaCl, 7 ​g L^-1^ KH_2_PO_4_, 3 ​g L^-1^ K_2_HPO_4_, 18 ​g ​L^-1^ glucose). Cell extracts were prepared by harvesting 50-mL cultures grown in baffled Erlenmeyer flasks to an OD600 of 5.0. Cells were harvested by centrifugation at 5000×*g* for 10 ​min in 50 ​mL volumes and washed twice with S30 buffer (14 ​mM magnesium acetate, 60 ​mM potassium acetate, 1 ​mM dithiothreitol (DTT) and 10 ​mM Tris-acetate, pH 8.2) by resuspension and centrifugation. The pellets were weighed, flash-frozen, and stored at −80 ​°C. Extracts were prepared by thawing and resuspending the cells in 0.8 ​mL of S30 buffer per gram of cell wet weight. The resuspension was lysed using 530 ​J per mL of suspension at 50% tip amplitude with ice water cooling. Following sonication, tubes of cell extract were centrifuged twice at 21,100×*g* for 10 ​min at 4 ​°C, aliquoted, frozen with liquid nitrogen, and stored at −80 ​°C.

### 2.3 Cell-free extract depletions

Cell extracts were depleted for specific proteins by adding one volume of cell extract to 0.2X volume of ice-cold HisPur™ Cobalt Resin (ThermoFisher Scientific) suspension in 1.5 ​mL microcentrifuge tubes. Prior to the addition of lysate, HisPur™ Cobalt Resin was washed 2X with 500 ​μL S30 buffer and incubated with 10 ​mM imidazole buffer (pH 4.5; 10 ​mM imidazole, 50 ​mM monopotassium chloride, 300 ​mM NaCl). Lysate-resin mixtures were incubated for 1 ​h at 4 ​°C under shaking conditions (800 ​rpm) to ensure the suspension of the resin particles in the extracts and then centrifuged at 14,000×*g* for 30 ​s. Supernatants were aliquoted, flash-frozen, and stored at −80 ​°C until used. His-tagged proteins were eluted from the HisPur™ Cobalt Resin by suspending the resin in 50 ​μL elution buffer (pH 4.5; 250 ​mM imidazole, 50 ​mM monosodium phosphate, 300 ​mM NaCl) for 30 ​min at 4 ​°C under shaking conditions (800 ​rpm). The eluate was obtained for proteomic quantification by spinning down the suspension at 14,000×*g* for 30 ​s and collecting the supernatant. The selective depletions were verified with an anti-6xHis Western Blot.

### 2.4 CFME reaction set-up

Glucose consumption reactions were carried out at 37 ​°C in 50 ​μL volumes using a solution of 100 ​mM glucose, 18 ​mM magnesium glutamate, 15 ​mM ammonium glutamate, 0.2 ​mM Coenzyme A, 195 ​mM potassium glutamate, 1 ​mM ATP, 150 ​mM Bis-Tris, 1 ​mM NAD^+^, 10 ​mM dipotassium phosphate. Similarly, pyruvate fed reactions were carried out using the same conditions with the exception of 25 ​mM pyruvate being used in place of glucose. Extracts were added to a final protein concentration of 4.5 ​mg ​mL^−1^. Each reaction was quenched by the addition of 50 ​μL of 5% TCA. The supernatant was centrifuged at 11,000×*g* for 5 ​min and directly used for analytical measurements.

### 2.5 Proteomics sample preparation

Samples of both depleted and nondepleted versions of WT, 6xHis*-pflB,* 6xHis-2, 6xHis-3, and 6xHis-4 ​cell extracts were each prepared in triplicate as follows. Extracts were solubilized in 200 ​μL of 4% SDS in 100 ​mM Tris buffer, pH 8.0. Trichloroacetic acid was added to achieve a concentration of 20% (w/v). Samples were vortexed and incubated at 4 ​°C for 2 ​h followed by 10 ​min ​at −80 ​°C. Samples were then thawed on ice prior to centrifugation (~21,000 ​g) for 10 ​min ​at 4 ​°C to pellet precipitated proteins from the detergent and solutes. The supernatant was discarded, and samples were washed with 1 ​mL of ice-cold acetone. Pelleted proteins were then air-dried and resuspended in 100 ​μL of 8 ​M urea in 100 ​mM Tris buffer, pH 8.0. Proteins were reduced with 10 ​mM dithiothreitol incubated for 30 ​min and alkylated with 30 ​mM iodoacetamide for 15 ​min in the dark at room temperature. Proteins were digested with two separate and sequential aliquots of sequencing grade trypsin (Promega) of 1 ​μg. Samples were diluted to 4 ​M urea and digested for 3 ​h, followed by dilution to 2 ​M urea for overnight digestion. Samples were then adjusted to 0.1% trifluoroacetic acid and then desalted on Pierce peptide desalting spin columns (Thermo Scientific) as per manufacturer’s instructions. Samples were vacuum-dried with a SpeedVac (Thermo Scientific) and then resuspended in 50 ​μL of 0.1% formic acid. Peptide concentrations were then measured using a NanoDrop spectrophotometer (Thermo Scientific) and 2 ​μg of each sample was used for LC-MS measurement.

### 2.6 LC-MS/MS analysis

All samples were analyzed on a Q Exactive Plus mass spectrometer (Thermo Scientific) coupled with an automated Vanquish UHPLC system (Thermo Scientific). Peptides were separated on a triphasic precolumn (RP-SCX-RP; 100 ​μm inner diameter and 15 ​cm total length) coupled to an in-house-pulled nanospray emitter of 75 ​μm inner diameter packed with 25 ​cm of 1.7 ​μm of Kinetex C18 resin (Phenomenex). For each sample, a single 2 ​μg injection of peptides were loaded and analyzed across a salt cut of ammonium acetate (500 ​mM) followed by a 210 ​min split-flow (300 ​nL/min) organic gradient, wash, and re-equilibration: 0%–2% solvent B over 27 ​min, 2%–25% solvent B over 148 ​min, 25%–50% solvent B over 10 ​min, 50%–0% solvent B over 10 ​min, hold at 0% solvent B for 15 ​min. MS data were acquired with the Thermo Xcalibur software using the top 10 data-dependent acquisition.

### 2.7 Proteome database search

All MS/MS spectra collected were processed in Proteome Discoverer, version 2.3 with MS Amanda and Percolator. Spectral data were searched against the most recent *E. coli* reference proteome database from UniProt to which mutated sequences and common laboratory contaminants were appended. The following parameters were set up in MS Amanda to derive fully tryptic peptides: MS1 tolerance ​= ​5 ​ppm; MS2 tolerance ​= ​0.02 ​Da; missed cleavages ​= ​2; Carbamidomethyl (C, + 57.021 ​Da) as static modification; and oxidation (M, + 15.995 ​Da) as dynamic modifications. The Percolator false discovery rate threshold was set to 1% at the peptide-spectrum match and peptide levels. FDR-controlled peptides were then quantified according to the chromatographic area-under-the-curve and mapped to their respective proteins. Areas were summed to estimate protein-level abundance.

### 2.8 Proteomic data analysis

For differential abundance analysis of proteins, the protein table was exported from Proteome Discoverer. Proteins were filtered to remove stochastic sampling. All proteins present in 2 out of 3 biological replicates in any condition were considered valid for quantitative analysis. Data was log_2_ transformed, LOESS normalized between the biological replicates and mean-centered across all the conditions. Missing data were imputed by random numbers drawn from a normal distribution (width ​= ​0.3 and downshift ​= ​2.8 using Perseus software (http://www.perseus-framework.org). The resulting matrix was subjected to ANOVA and a post-hoc TukeyHSD test to assess protein abundance differences between the different experimental groups. The statistical analyses were done using an in-house developed R script.

### 2.9 Metabolite measurements

Glucose, pyruvate, lactate, acetate, formate, and ethanol measurements were performed via high-performance liquid chromatography (HPLC) with an Agilent 1260 equipped with an Aminex HPX 87-H column (Bio-Rad, Hercules, CA). Analytes were eluted with isocratic 5 ​mM sulfuric acid at a flow rate of 0.55 ​mL min^–1^ at 35 ​°C for 25 ​min. Metabolite concentrations were calculated from measurements collected through a refractive index detector (Agilent, Santa Clara, CA) and a diode array UV–visible detector (Agilent, Santa Clara, CA) reading at 191 ​nm. Pyruvate, glucose, lactate, acetate, formate, and ethanol standards were used for sample quantification using linear interpolation of external standard curves.

## 3 Results and discussion

**Fig. 2:**
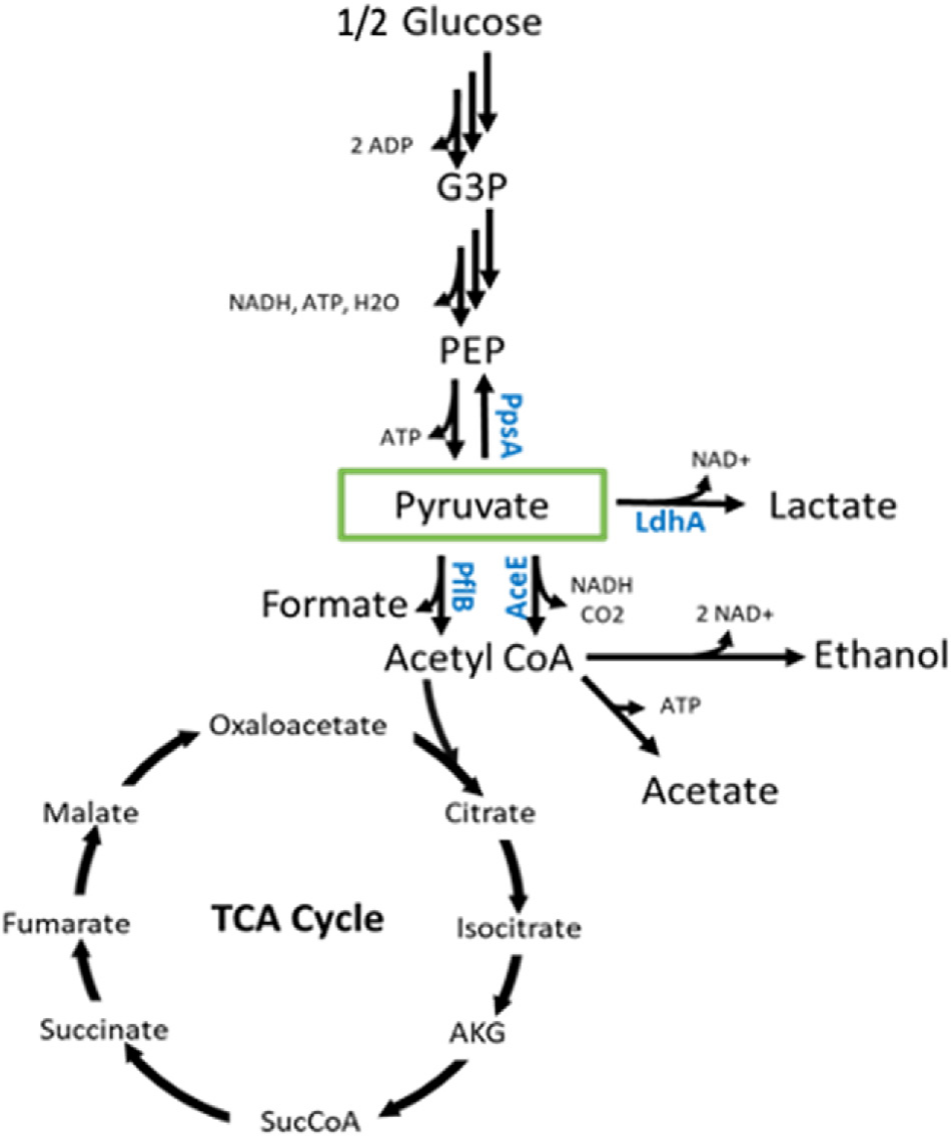
Glycolysis and engineered pathway nodes showing the location of the modified enzymes PflB, LdhA, PpsA, AceE.

Pyruvate sits at the biochemical junction of glycolysis and the TCA cycle. It is a key intermediate in producing many food, cosmetic, pharmaceutical, and agricultural products whose improved production has been largely unexplored in cell-free systems (Zhang et al., 2016; Maleki and Eiteman, 2017). In order to create a pyruvate pooling phenotype in an *E. coli* cell-free extract, four proteins were chosen as targets for removal, LdhA, PflB, AceE, and PpsA (Table 1) (Fig. 2) (Maleki and Eiteman, 2017). These were chosen due to their direct role in consuming pyruvate as well as the likelihood that they are active in lysates. LdhA was selected as the production of lactate from pyruvate has been observed in cell-free systems generated under similar conditions to those prepared here (Karim et al., 2020; Dudley et al., 2019). Since LdhA has previously been reported to be absent in lysates derived from aerobically grown cultures, we assumed that oxygen-limited zones are present in the cultures upon harvesting at mid-log phase (Bujara et al., 2011). This is typical for cells grown in flasks, even under constant shaking, in media with high concentrations of glucose such as the 2xYPTG media (18 ​g ​L^−1^ glucose) used here (Soini et al., 2008; Enfors et al., 2001). Under this assumption, PflB is also likely expressed minimally in the culture and would be capable of carrying pyruvate flux for glycolytic fermentation (Wang et al., 2010; Xu et al., 1999). At the same time, pyruvate dehydrogenase (Pdh), responsible for pyruvate flux in aerobic conditions, is also expected to be expressed under these conditions as respiratory metabolism is reportedly active in S30 lysates (Foshaget al., 2018). Pdh expressed in oxygen-limited cultures is additionally known to be functional in *E. coli* lysates as long as NADH concentrations are not allosterically inhibiting (Snoep et al., 1993; Murarka et al., 2010). During a cell-free metabolic reaction, one might expect PflB to be inactivated by oxygen due to its oxygen-sensitivity, and for pyruvate to acetyl-CoA flux to be controlled by Pdh (Wagner et al., 1992). Such activity however has yet to be reported so both PflB and AceE, the E1 component of Pdh, were additionally chosen as targets here. We also selected PpsA under the assumption that back-flux to phosphoenolpyruvate (PEP) might occur upon high pyruvate pooling in the lysates (Fig. 2). 6xHis tags were fused on either the amino or carboxyl terminus by genetic modification based on an evaluation of literature related to previous purification attempts or crystal structures in order to find a non-inhibitory location (Table 1) (Lougheed et al., 2014; Halliwell et al., 2001).

**Table 1:** Gene and protein information for MAGE targets with a potential effect on pyruvate metabolism.

Gene	Protein	6xHis-Tagged Terminus	MW (kDa)
pfIB	Pyruvate formate-lyase	N-Terminal	85
IdhA	D-lactate dehydrogenase	N-Terminal	36.53
ppsA	Phosphoenolpyruvate synthase	N-Terminal	87.43
aceE	Pyruvate dehydrogenase E1	C-Terminal	99.66

**Fig. 3:**
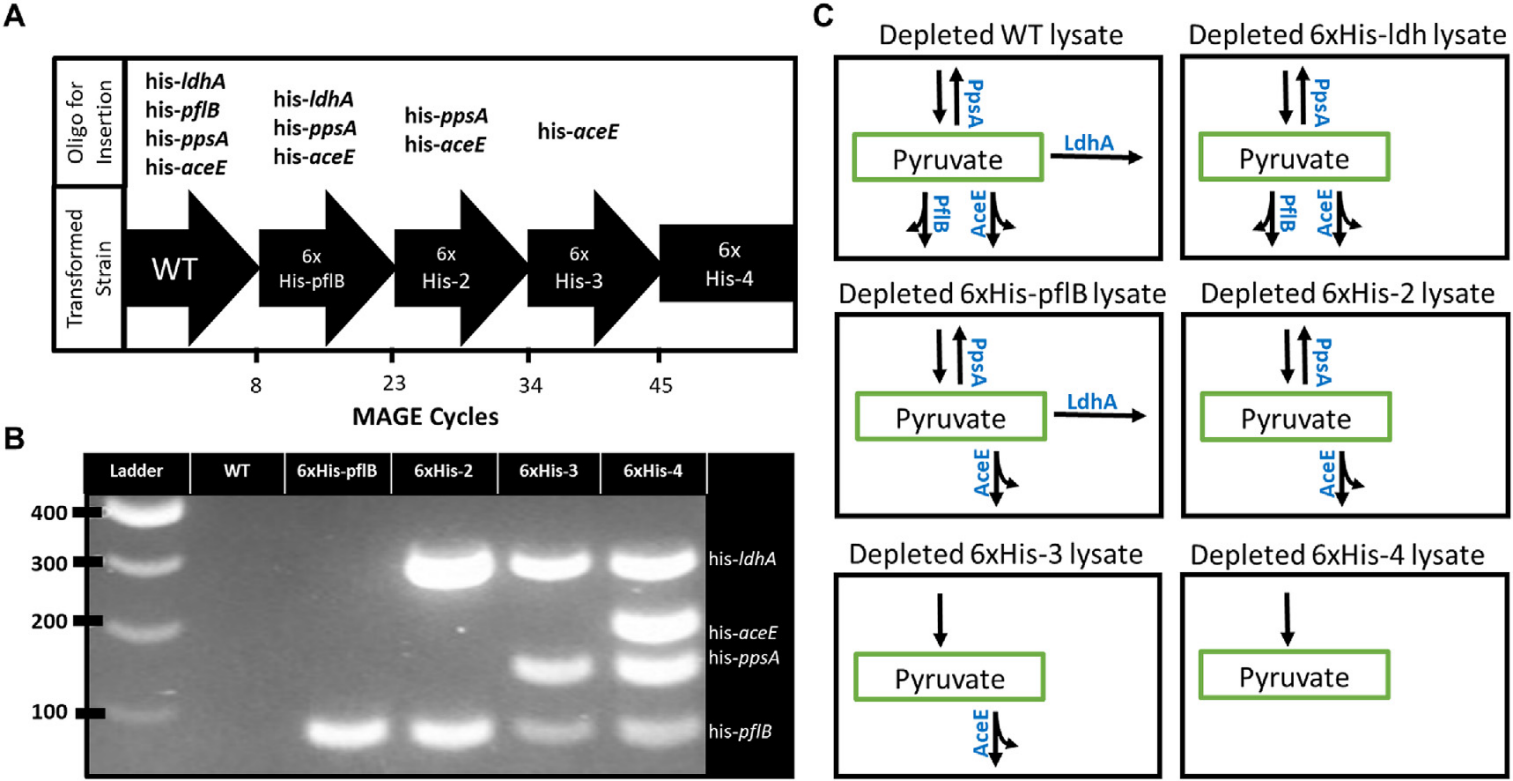
Source strain multiplex genome engineering and expected metabolic phenotypes of derived lysates post-depletion. **A.** Strain construction course by MAGE cycling culminating with the 6xHis-4 containing all 4 tags. Each arrow designates the strain being taken through the MAGE process with the oligos used to transform each strain above the arrow. **B.** MASC-PCR results for additive mutations using primers specifically designed for the 6xHis-tagged version of the gene. **C.** Expected metabolic phenotypes present in WT and engineered lysate proteomes after the depletion of lysates derived from all generated strains.

The pORTMAGE system was used instead of the traditional genome integrated system due to its potential transferability to multiple donor organisms including *E. coli* BL21 Star(DE3) (Wang et al., 2009; Ronda et al., 2016). Additionally, pORTMAGE is curable following genome engineering and relieves the metabolic burden on the cell that can be imparted due to plasmid maintenance (Silva et al., 2012). Colony screening was performed using MASC-PCR and further verified using Sanger sequencing (Fig. 3B) (Wang and Church, 2011). A total of 5 strains were used for this work. (Table 2). The strains included 6xHis-*pflB*, 6xHis-2 (6xHis-*pflB-ldhA*), 6xHis-3 (6xHis-*pflB-ldhA-ppsA*), 6xHis-4 (6xHis-*pflB-ldhA-ppsA-aceE*) and 6xHis-*ldhA,* and their lysates were expected to have varying metabolic phenotypes post-depletion (Fig. 3C). Sixty rounds of MAGE were needed to incorporate all four of the tags into the *E. coli* genome (Fig. 3A) (Table 2). This is high when compared to studies producing single nucleotide changes but in line with other efforts using 6xHis-tags with a genome incorporated MAGE system (Wang et al., 2012).

**Table 2:** Strains created for this study.

Strain Name	Background	Modified Genes
6xHis-*pflb*	BL21 Star(DE3)	6xhis-*pflB*
6xHis-*ldhA*	BL21 Star(DE3)	6xhis-*ldhA*
6xHis-2	BL21 Star(DE3)	6xhis-*ldhA,* 6xhis-*pflB*
6xHis-3	BL21 Star(DE3)	6xhis-*ldhA,* 6xhis-*pflB,* 6xhis-*ppsa*
6xHis-4	BL21 Star(DE3)	6xhis-*ldhA,* 6xhis-*pflB,* 6xhis-*ppsa,* 6xhis-*aceE*

After curing each strain of the pORTMAGE plasmid, potential inhibitory effects on growth caused by the expression of tagged proteins were evaluated. Though the presence of the polyhistidine tags has previously been observed to cause growth defects due to the stability of tagged proteins, none of the cells produced for this work showed a significant drop in growth rate (Supplemental Figure 1) (Booth et al., 2018; Wingfield, 2015).

**Fig. 4:**
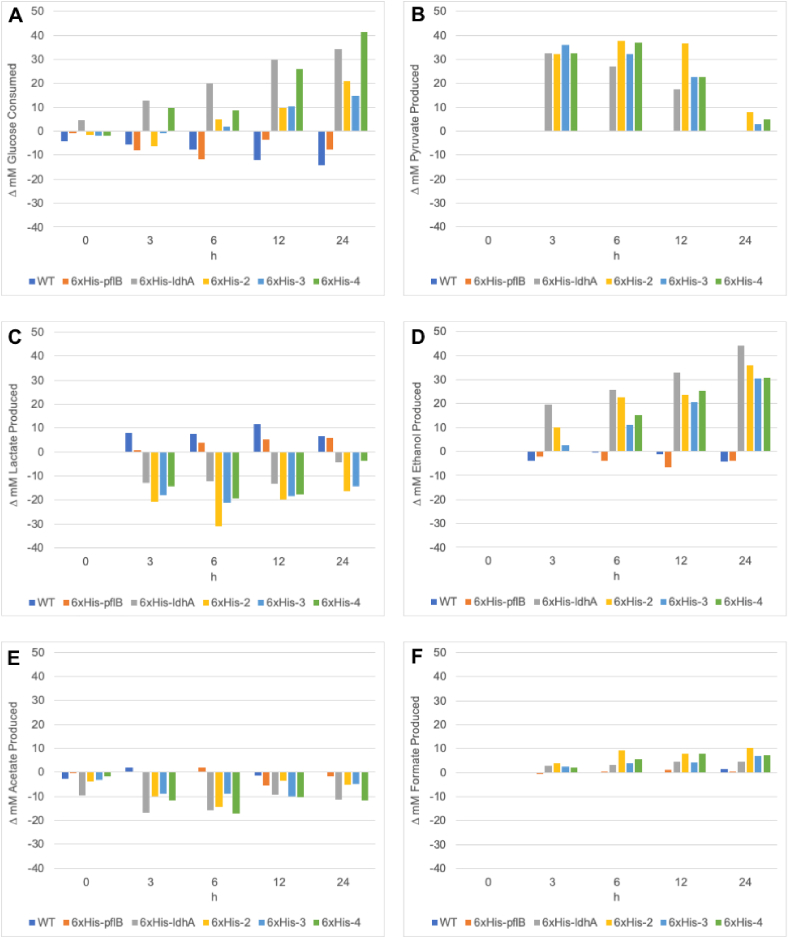
Relative changes between nondepleted and depleted versions of the lysates in terms of **A.** glucose consumption (nondepleted minus depleted), and **B.** pyruvate, **C.** lactate, **D.** ethanol, **E.** acetate, and **F.** formate production (depleted minus nondepleted) over time. Depleted extracts have had specific 6xHis-tagged proteins removed by incubation with cobalt beads. Extracts containing tagged proteins, but without an incubation step, are referred to as nondepleted. Data for the time course reactions were acquired using n ​= ​3 biological replicates. Standard errors calculated for replicates were negligible and are visualized in Supplemental Figure 2.

The effect of proteome reduction on the extract’s metabolic profile was then tested by measurement of glucose consumption, pyruvate accumulation, and the pooling of fermentation end-products (i.e., lactate, ethanol, formate, and acetate) in a CFME reaction mix. As nonspecific binding is commonly associated with the use of 6xHis-tags, we evaluated whether the reduction method would result in significant alterations in lysate metabolism. Evidently, the wild-type derived lysate and the wild-type lysate taken through the depletion process have comparable glucose consumption and fermentation end-product pooling (Supplemental Figure 2A). Initial concentrations of acetate from the S30 buffer used to prepare the extracts could be observed (Supplemental Figure 2). Further, there is no apparent pyruvate accumulation after incubation of the WT lysates with cobalt beads, indicating that the depletion process does not remove proteins that affect cell-free pyruvate production in an appreciable manner (Supplemental Figure 2A). Extracts derived from 6xHis-*pflB*, 6xHis-*ldhA,* 6xHis-2, 6xHis-3, and 6xHis-4 were thus reduced and assessed for glucose consumption and pyruvate build-up relative to their unreduced counterparts (Fig. 4A and B). Significantly, none of the nondepleted extracts derived from these strains accumulated pyruvate, and metabolite profiles all trended similarly in terms of glucose consumption and fermentation end-product pooling (Supplemental Figure 2). A noteworthy general observation from the metabolite profiles of the depleted 6xHis-*pflB*, 6xHis-*ldhA*, 6xHis-2, 6xHis-3, and 6xHis-4 lysates is that they all continue to accumulate downstream products of the target pyruvate-consuming enzymes, albeit with varying trends (Supplemental Fig. 2B–2F). This would suggest that the depletion method did not completely remove the targeted enzymes from the lysate proteome, but evidently allows a degree of targeted protein depletion that results in significant metabolic changes.

The targeted depletion of PflB from the 6xHis-*pflB* extract results in a metabolic profile that is similar to its control counterpart in that neither accumulate pyruvate (Fig. 4B). Changes in glucose consumption and lactate, ethanol, acetate, and formate production between the depleted 6xHis-*pflB* and WT lysates, relative to their nondepleted controls, are also insignificant (Figure 4A, 4C–4F). This metabolic phenotype could be expected considering that Pdh activity is active in crude extracts of *E. coli* (Snoep et al., 1993). The Pdh complex has a higher affinity for pyruvate than PflB (*K*_*m*_ ​= ​0.4 versus 2.0 ​mM, respectively) when its activity is not inhibited by NADH such as in the presence of an NADH sink like LdhA (Fig. 2) (Wang et al., 2010). Thus, regardless of whether PflB concentrations are decreased, Pdh could likely be an active route for flux towards ethanol and acetate in this lysate.

The lysate with targeted depletions of both PflB and LdhA (6xHis-2, depleted) pooled 32 ​mM pyruvate relative to its nondepleted control in 3 ​h (Fig. 4B). This lysate additionally consumed glucose steadily while maintaining a > 30 ​mM pyruvate concentration until 12 ​h. The depletion of LdhA is supported by the observation of a lower lactate concentration in reactions run in these lysates compared to their nondepleted counterparts (Fig. 4C). The rapid build-up of ethanol (20 ​mM in 3 ​h relative to the control) in these lysates, and the likely increased activation of aldehyde-alcohol dehydrogenase (AdhE) as an alternative sink for NAD^+^ regeneration, also supports successful LdhA depletion (Figs. 2 and 4D)**.** Acetate production is also not observed after LdhA depletion, presumably pointing to the increased funneling of acetyl-CoA from the Pdh reaction towards ethanol production to meet redox balance (Fig. 4E). Rather, acetate seems to be increasingly consumed as a secondary carbon source likely for generating more acetyl-CoA through the acetyl-CoA synthetase route (Fig. 4E) (Kumari et al., 2000). On the other hand, the contribution of depleted PflB to the observed metabolic phenotype in reactions run in this 6xHis-2 lysate is not as immediately observable. However, at time points before 12 ​h, there is a notable decrease in lactate and increased maintenance of high pyruvate concentrations (Fig. 4B and C). In comparison, the depleted 6xHis-*ldhA* lysate was not as efficient at retaining high pyruvate concentrations as the depleted 6xHis-2 lysate (Fig. 4B). The depletion of both LdhA and PflB from the 6xHis-2 extract may funnel pyruvate flux through Pdh, a claim bolstered by previous work showing NADH-insensitive Pdh to limit glucose fermentation in the absence of both PflB and LdhA (Wang et al., 2010). Thus, the pyruvate pooled up to 12 ​h in the depleted 6xHis-2 lysate likely results from lowered glycolytic rates in these extracts. This interpretation is supported by the lowered consumption of glucose in the 6xHis-2 lysate compared to the 6xHis-*ldhA* lysate (Fig. 4A). The concomitant increase in ethanol production and significantly lowered lactate synthesis at 3 ​h and 6 ​h in the depleted lysate relative to its control additionally suggests that pyruvate flux is directed through Pdh-AdhE maintaining redox balance by generating net 1 ​mol NAD^+^ per mol pyruvate (Fig. 4C and D) (Wang et al., 2010). Compared to reactions run when depleting PflB and LdhA individually, the co-depletion of LdhA and PflB has an additive effect on cell-free pyruvate pooling. This interpretation suggests that oxygen-sensitive PflB is indeed active in *E. coli* crude extracts, which is supported by the observable production of formate after LdhA reductions (Fig. 4F). We reason that decreasing the concentrations of the NAD^+^ regenerating LdhA enzyme limits the *in vitro* activity of formate dehydrogenases that require NAD^+^ as a substrate to decompose formate to CO_2_ and H_2_O, thus resulting in formate build-up (Raaijmakers and Romão, 2006).

Compared to the depleted 6xHis-2 lysate, the pull-down of PpsA from the 6xHis-3 lysate led to a steady decrease of the pyruvate concentration after 3 ​h (Fig. 4B). This observation presumably points to the importance of PpsA as an ATP sink in *in vitro* metabolism. *E. coli* glycolytic flux is naturally responsive to the cell’s energy charge via the allosteric inhibition of phosphofructokinase and pyruvate kinase by ATP (Waygood et al., 1976; Baez et al., 2013). The build-up of ATP in the depleted 6xHis-3 lysate that results from glycolysis can lead to lower pyruvate production from glucose at later timepoints, a claim additionally supported by the decrease in relative glucose consumed at 24 ​h between the 6xHis-2 and 6xHis-3 lysates (Fig. 4A). We additionally observed lower productivities (15 ​mM in 3 ​h) and final titers (41 ​mM) of ethanol formation in the depleted 6xHis-3 extract compared to reactions run in the depleted 6xHis-2 lysate (productivity ​= ​31 ​mM in 3 ​h; titer ​= ​55 ​mM) (Fig. 4D, Supplemental Figure 2C and 2D). The low initial accumulation of ethanol despite high pyruvate pooling from LdhA depletion is possibly due to decreased Pdh activity under high adenylate charge (Shen and Atkinson, 1970). The same condition can explain lowered ethanol production in the depleted 6xHis-3 extract compared to the depleted 6xHis-2 lysate (Fig. 4D). Alternatively, the less efficient ethanol pooling can be due to lowered synthesis rates of NADH from glycolysis after PpsA pull-down.

The targeted depletion of AceE, a component of Pdh, did not increase pyruvate pooling capabilities but led to the highest consumption of glucose observed (Fig. 4A, Supplemental Figure 2F). We reason that perturbing the redox balance in the lysate through the pull-down of AceE and thus the depletion of NAD^+^-dependent Pdh activity increased the availability of NAD^+^ for increased glycolytic flux (Fig. 2) (Zhu et al., 2008). Moreover, the depletion of Pdh activity seems to shift the maintenance of redox balance back to LdhA at later timepoints, as suggested by the steady increase in lactate levels with the decrease in glucose stores (Fig. 4A and C). NAD^+^ is thus continually regenerated by remaining LdhA and ethanol production for the NADH generating step in glycolysis, but this could possibly result in the build-up of glycolytic intermediates since the total consumption of 100 ​mM glucose is not fully accounted for by the concentrations of pooled fermentation end-products. In general, the rapid consumption of the NAD^+^ supply could be limiting due to the potential lack of cofactor recycling initiated by the decrease of LdhA levels. Pyruvate consumption experiments performed with the WT and 6xHis-4 lysates show that a significant portion of the pyruvate consuming pathways remain robust after reduction evidencing that the constraint may be due to bottlenecks in upstream glycolysis and further shows that a balance between glucose and pyruvate consumption leads to the engineered pyruvate pooling phenotype (Fig. 4B, Supplemental Figure 3).

**Fig. 5:**
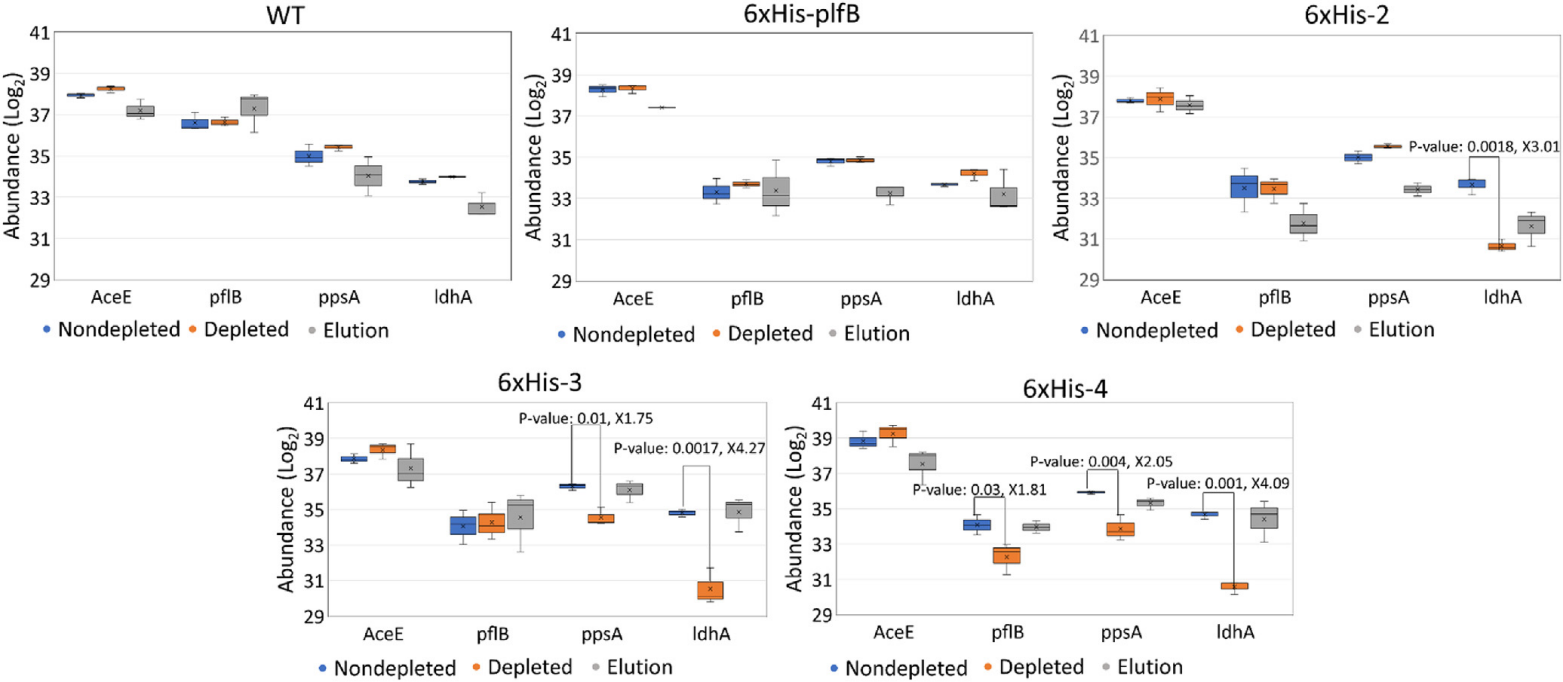
Proteomic analysis of control cell-free extracts without depletion (blue), and a depleted cell-free extract (orange), and the elutions from the depleted extracts (gray). Significant fold-changes in protein concentration when comparing the depleted to the nondepleted extract are denoted by p-value and fold change reduction in concentration of the protein above a bracket. Panels A, B, C, D, and E denote the WT, 6xHis-pflB, 6xHis-2, 6xHis-3, and D. 6xHis-4 strains, respectively. Asterisks indicate proteins targeted for removal in the depleted strain, each experiment is derived from n ​= ​3 biological replicates. (For interpretation of the references to colour in this figure legend, the reader is referred to the Web version of this article.)

From the mass spectrometry-based proteomics profiling, it is evident that 6xHis-tagged LdhA and PpsA could be removed from lysates, while significant removal of 6xHis-tagged PflB was not successfully detected (Fig. 5B–E). Although the decrease in PflB levels between the nondepleted and depleted 6xHis-4 lysates met the significance threshold (pval ​< ​0.05), the change was only a 1.81-fold reduction compared to the significant decreases in LdhA and PpsA levels following lysate depletion (Fig. 5E). AceE was not observed to be pulled down after the purification of 6xHis-tagged proteins from the 6xHis-4 lysate. These findings are inconsistent with metabolic output data as the depletion of 6xHis-4 lysate causes a more significant glucose consumption phenotype than extracts with fewer tags (Fig. 4A). However, anti-6xHis western blots of eluants from the cobalt beads used to deplete the engineered extracts show an enrichment for each of the targeted proteins in their respective strains while no enrichment was seen in the elution from the WT (Supplemental Figure 4). The corroborating evidence of the targeted metabolite analyses combined with antibody tagging leads us to conclude that the targeted proteins are being sufficiently removed and affect the reactivity of the extracts. The inconsistency in the results obtained from mass spectrometry and western blot analyses can be explained away as differences between the analytical techniques. Mass spectrometry is recognized for its ability to identify and quantify proteins in complex sample mixtures and for doing so with higher reliability, reproducibility, specificity, and sensitivity when compared to western blots (Aebersold et al., 2013; Jayasena et al., 2016). Here, the comprehensive, MS-based proteomic analyses involve different sample types, the nondepleted lysates, depleted lysates, and the eluants, and the different background signals may complicate comparisons (Kim et al., 2015). In contrast, the western blot experiment focuses on the analysis of a specific protein in the eluant protein fraction. These techniques complement each other and highlight the different strengths of the two approaches. Whereas the western blot provides confirmation of protein removal in the relatively simple eluant, it lacks the quantitative rigor of mass spectrometry that is needed for comprehensive analyses of complex samples. Therefore, the western blot provides an orthogonal complement to the MS-based results and provides support for the observed metabolic output data. While western blot analysis validated the depletion of proteins not identified through comparative mass spectrometry analysis, future efforts can benefit from a more targeted proteomics approach using labeled peptides to determine absolute quantitative measurements of the method’s depletion efficiency for targeted proteins.

Nonetheless, our comparative mass spectrometry analysis provided additional information about our method. The results show that the incorporation of 6xHis-tags into the genomes had minimal effects on the expression of pyruvate-consuming enzymes in all strains’ proteomes (Fig. 5, blue boxes), allowing the pull-down of one target protein without altering the concentrations of other pyruvate-consuming enzymes. This observation is corroborated by the comparable trends among the metabolite profiles of all nondepleted lysates (Supplemental Figure 2). This advances our method for precisely generating unconventional metabolic phenotypes that cannot be achieved via gene deletions, since knock-outs of metabolic genes would incite global proteomic and thus metabolic changes in cells (Kabir et al., 2005). We further analyzed relative proteomic changes in the nondepleted and depleted extracts to determine whether the method resulted in the removal of off-target proteins. Importantly, the process of depleting the proteome did not seem to significantly impact proteins in central metabolism outside of those targeted by the tagging process. When comparing the depleted and nondepleted WT lysates, in the 58 proteins with a greater than 4-fold reduction, none were connected to central metabolism outside of roles in membrane transport (Supplemental Data 1). Future efforts will seek to minimize off-target effects in order to improve the general applicability of lysate proteome engineering. Though outside the initial scope of this study as the main prospect was to show the use of enzyme depletion as a tool for CFME, targeted proteomics could be an effective tool to connect concentrations of depleted proteins with their resultant metabolic profile.

Targeted depletion of a lysate proteome enables a rapid means to manipulate central metabolism without the possible drawback of cultivating “sick” organisms as often results from traditional, *in vivo* metabolic engineering efforts. The pORTMAGE system offers the potential for extension of this engineering strategy to other, non-model cell-free chassis. Though not all proteins targeted for depletion could be shown to be depleted in substantial quantities through proteomics, our analysis of the metabolic products and western blot analysis shows clear differences between the extracts following each tagging and only following depletion. In contrast with gene knockout strategies that result in global proteomic changes during source strain cultivation, this method allows removal of selected proteins from a lysate proteomic background that is similar to the wild type derived extracts, allowing targeted manipulation of lysate proteomes. Thus, although lysates derived from the deletion of a target gene or the post-lysis depletion of its corresponding protein are expected to have different metabolic phenotypes, our CFME approach could be broadly applied to yield metabolic states that are not traditionally possible in living organisms. Future improvements to lysate proteome engineering could make use of multiplex genome engineering methods that are amenable to the insertion of larger tags as MAGE based methods are naturally limited to low-base pair insertions (Ronda et al., 2016; Dalia et al., 2017; Mosberg et al., 2010). To further advance the depletion of specific proteins in the lysate’s proteome, orthogonal protein degradation systems could be employed wherein proteins are genomically tagged and degraded in a cell-free extract using an exogenous protease. The *mf-lon* protease system serves this function through a 27 amino acid long peptide and could allow for titration experiments leading to complete degradation of the proteins of interest (Gur and Sauer, 2008; Cameron and Collins, 2014). A key factor to note stems from MAGE’s limited throughput when making large additions to the genome. Whereas single base changes can be added with ease, longer tags such as 6xHis tags, are near the edge of feasibility for MAGE tagging. Organisms such as *Vibrio natriegens* can take advantage of a MAGE like process termed MUGENT that allows for significantly longer incorporations at the cost of using a donor strain with less study than *E. coli* (Dalia et al., 2017; Wiegand et al., 2019).

## 4 Conclusions

Shown in this work was the use of genome engineering to create protein modifications that allow for the control of metabolic activity in cellular lysates. This cell-free metabolic engineering strategy allows for the targeted removal of enzymes that can enable the focused production of metabolites from simple precursors using rapidly prepared crude extracts that would otherwise lead to changes in metabolic state and significant growth defects in living cells (Kabir et al., 2005; Zhou et al., 2011; Richards et al., 2013; Schutte et al., 2015). The ability to extract pyruvate degrading enzymes, leading to unconventional metabolic states, was engineered and shown to be capable of pooling pyruvate for a significant period of time as well as improving the ethanol titer of the extract. The ability to direct metabolic flux in cell-free systems and create proteomes untenable to living cells was demonstrated. The flexibility of CFME systems highlights the significant value they hold as novel bioproduction platforms. The advances made in this work can be extended to design molecule specific donor strains for natural product biosynthesis, such as for polyketides or carbohydrates, through the removal of defined inhibitory reactions. The removal of specific components of crude lysates allows for more complex reaction networks to be employed in the development of CFME bioproduction platforms. As CFME begins to tackle new challenges related to antibiotic, fuel, and, materials production, innovative engineering tools and techniques designed to improve its efficiency will be crucial to advancing the scope and adoption of cell-free biological production.

## CRediT authorship contribution statement

**David C. Garcia:** Conceptualization, Data curation, Formal analysis, Investigation, Methodology, Validation, Visualization, Writing - original draft, Writing - review & editing. **Jaime Lorenzo N. Dinglasan:** Conceptualization, Data curation, Formal analysis, Investigation, Methodology, Validation, Visualization, Writing - original draft, Writing - review & editing. **Him Shrestha:** Conceptualization, Data curation, Formal analysis, Methodology, Writing - original draft, Writing - review & editing. **Paul E. Abraham:** Methodology, Funding acquisition, Project administration, Resources, Supervision, Writing - review & editing. **Robert L. Hettich:** Methodology, Funding acquisition, Project administration, Resources, Supervision, Writing - review & editing. **Mitchel J. Doktycz:** Conceptualization, Methodology, Funding acquisition, Project administration, Resources, Supervision, Visualization, Writing - review & editing.

## Declaration of competing interest

The authors declare that they have no known competing financial interests or personal relationships that could have appeared to influence the work reported in this paper.
