# Supplementary material for: A lysate proteome engineering strategy for enhancing cell-free metabolite production
Source: Metab Eng Commun. 2021 Jan 22;12:e00162. doi: 10.1016/j.mec.2021.e00162 (PMC7851839; doi:10.1016/j.mec.2021.e00162)
Supplement: Multimedia component 1 [file mmc1.docx]

**Supplemental Material:**

**Supplemental Table 1.** MAGE oligos use for this study. Phosphorothioated bases are noted with asterisks.

| Primer | Sequence |
| --- | --- |
| Pfl | a*a*t*a*aaaaatccacttaagaaggtaggtgttacatgCACcatCACcatCACCATtccgagcttaatgaaaagttagccacagcctgggaa |
| Ldh | t*a*a*a*tgtgattcaacatcactggagaaagtcttatgCACcatCACcatCACCATaaactcgccgtttatagcacaaaacagtacgacaag |
| Ppsa | c*a*a*a*ccgttcatttatcacaaaaggattgttcgatgCACcatCACcatCACCATtccaacaatggctcgtcaccgctggtgctttggtat |
| Pdh | a*c*t*c*aacgttattagatagataaggaataacccatgCACcatCACcatCACCATtcagaacgtttcccaaatgacgtggatccgatcgaa |

**Supplemental Table 2.** MASC-PCR oligos used for this study.

| Primer | Sequence |
| --- | --- |
| Pfl F | GCCAGCCAGGAAGGACTCGTCACCCTCG |
| Pfl R | GCAGTAAATAAAAAATCCACTTAAGAAGGTAGGTGTTACATGC |
| Ldh F | CAGCGTCATCATCATACCGATGGC |
| Ldh R | CTTAAATGTGATTCAACATCACTGGAGAAAGTCTTATGC |
| Ppsa F | GCTGGTTTACGCCGCTTTGGTCC |
| Ppsa R | ACCGTTCATTTATCACAAAAGGATTGTTCGATGC |
| Pdh F | TGGCCTTTATCGAAGAAATTTTGCTCGACAG |
| Pdh R | ATCCACGTCATTTGGGAAACGTTCTGAA |

**Supplemental Figure 1.** Growth rate and terminal OD600 as measured using a WPA CO 8000 Cell Density Meter (Biochrom Ltd., Cambridge, UK) for each of the four main strains generated in this study.


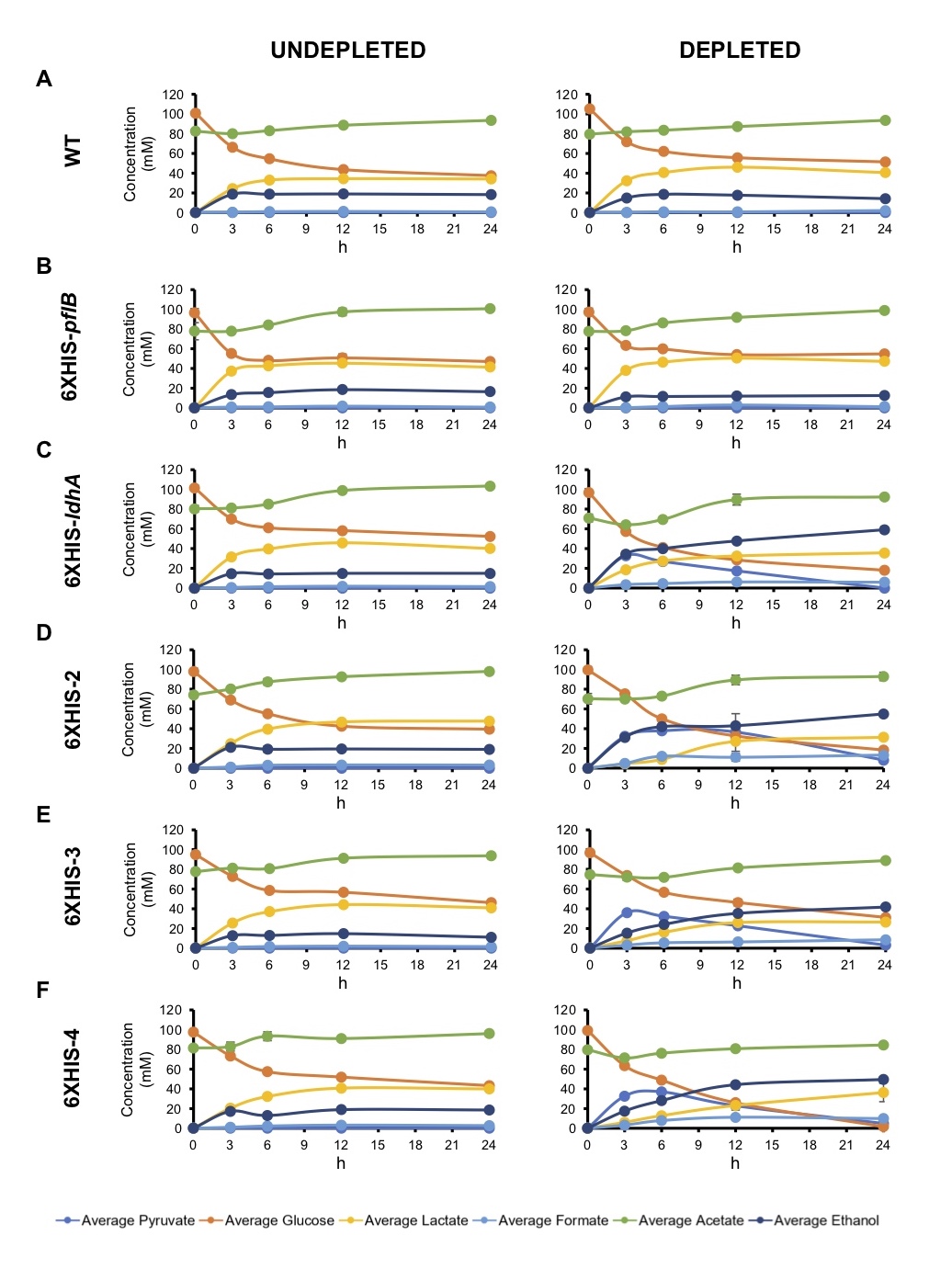


**NONDEPLETED**

**DEPLETED**

**Supplemental Figure 2.** Glucose consumption and fermentation product concentrations over time in A. WT, B. 6xHis-pflB, C. 6xHis-ldhA, D. 6xHis-2, E. 6xHis-3, and F. 6xHis-4 lysates. Data and standard deviation for the time course reactions were acquired using n=3 biological replicates. Depleted extracts have had specific 6xHis-tagged proteins removed by incubating them with cobalt beads. Extracts containing tagged proteins, but without an incubation step, are referred to as nondepleted.

6xHis-4 Depleted

WT-Depleted

**Supplemental Figure 3.** Comparison of pyruvate consumption using depleted WT and Δ4 extracts. All reaction conditions were exactly as those used for the glucose consuming reactions aside from the replacement of glucose with 25 mM pyruvate.

**
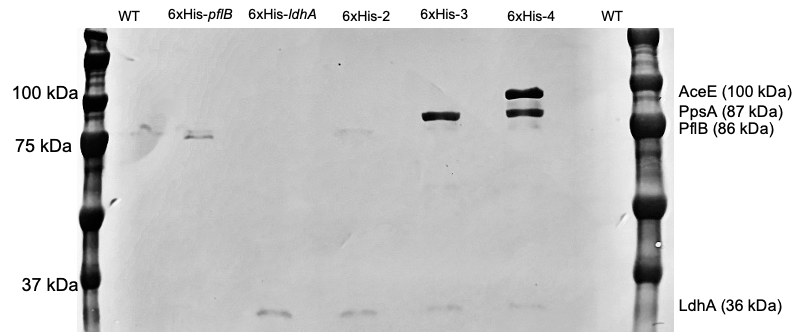
**

**Supplemental Figure 4.** Anti-6xHis western blot showing successful pull-downs of target proteins. Briefly, eluants from cobalt beads used to treat each lysate were concentrated to 0.6 mg/mL total protein concentrations. Protein solutions were denatured with equal volumes of 2x Laemli Sample Buffer (Bio-Rad) and boiled. Samples were loaded onto a 10 % Tris-glycine gel (Bio-RAD) and separated by denaturing PAGE. A Bio-Rad (Hercules, California) Trans-Blot SD Semidry Transfer Cell was used to transfer bands onto a PVDF membrane. The membrane was probed with 6xHis-tag Monoclonal Antibodies (Thermo Fisher Scientific, Waltham, MA) overnight after blocking with Pierce Protein Free (TBS) Blocking Buffer (Thermo Fisher Scientific, Waltham, MA). Blots were incubated with the relevant secondary antibody, HRP conjugate then developed with Pierce DAB Substrate (Thermo Fisher Scientific, Waltham, MA).
